# Insights into the Effects and Mechanism of Andrographolide-Mediated Recovery of Susceptibility of Methicillin-Resistant Staphylococcus aureus to β-Lactam Antibiotics

**DOI:** 10.1128/spectrum.02978-22

**Published:** 2023-01-05

**Authors:** Qiang Ma, Guilai Wang, Na Li, Xin Wang, Xinyun Kang, Yanni Mao, Guiqin Wang

**Affiliations:** a Veterinary Pharmacology Lab, College of Agriculture, Ningxia University, Yinchuan, Ningxia, China; b Yinchuan Hospital of Traditional Chinese Medicine, Yinchuan, Ningxia, China; University of Guelph

**Keywords:** andrographolide, methicillin-resistant *Staphylococcus aureus*, β-lactam antibiotics, β-lactamase, inhibitor

## Abstract

The frequent resistance associated with β-lactam antibiotics and the high frequency of mutations in β-lactamases constitute a major clinical challenge that can no longer be ignored. Andrographolide (AP), a natural active compound, has been shown to restore susceptibility to β-lactam antibiotics. Fluorescence quenching and molecular simulation showed that AP quenched the intrinsic fluorescence of β-lactamase BlaZ and stably bound to the residues in the catalytic cavity of BlaZ. Of note, AP was found to reduce the stability of the cell wall (CW) in methicillin-resistant Staphylococcus aureus (MRSA), and in combination with penicillin G (PEN), it significantly induced CW roughness and dispersion and even caused its disintegration, while the same concentration of PEN did not. In addition, transcriptome sequencing revealed that AP induced a significant stress response and increased peptidoglycan (PG) synthesis but disrupted its cross-linking, and it repressed the expression of critical genes such as *mecA*, *blaZ*, and *sarA*. We also validated these findings by quantitative reverse transcription-PCR (qRT-PCR). Association analysis using the GEO database showed that the alterations caused by AP were similar to those caused by mutations in the *sarA* gene. In summary, AP was able to restore the susceptibility of MRSA to β-lactam antibiotics, mainly by inhibiting the β-lactamase BlaZ, by downregulating the expression of critical resistance genes such as *mecA* and *blaZ*, and by disrupting CW homeostasis. In addition, restoration of susceptibility to antibiotics could be achieved by inhibiting the global regulator SarA, providing an effective solution to alleviate the problem of bacterial resistance.

**IMPORTANCE** Increasingly, alternatives to antibiotics are being used to mitigate the rapid onset and development of bacterial resistance, and the combination of natural compounds with traditional antibiotics has become an effective therapeutic strategy. Therefore, we attempted to discover more mechanisms to restore susceptibility and effective dosing strategies. Andrographolide (AP), as a natural active ingredient, can mediate recovery of susceptibility of MRSA to β-lactam antibiotics. AP bound stably to the β-lactamase BlaZ and impaired its hydrolytic activity. Notably, AP was able to downregulate the expression of critical resistance genes such as *mecA*, *blaZ*, and *sarA*. Meanwhile, it disrupted the CW cross-linking and homeostasis, while the same concentration of penicillin could not. The multiple inhibitory effect of AP resensitizes intrinsically resistant bacteria to β-lactam antibiotics, effectively prolonging the use cycle of these antibiotics and providing an effective solution to reduce the dosage of antibiotics and providing a theoretical reference for the prevention and control of MRSA.

## INTRODUCTION

The primary mechanism of bacterial resistance to β-lactam antibiotics is through the expression of β-lactamase, which hydrolyzes drug molecules (1). Studies on this class of drugs have also focused on inducing tolerance to β-lactamases and enhancement of antimicrobial activity. To date, more than 220 mutants of class A β-lactamases alone have been identified, and mutations in a few residues have been reported to cause 10-fold changes in its hydrolytic activity (2–4). Therefore, studies focusing on bacterial resistance should no longer be limited to the presence or absence of β-lactamases but should also begin to focus on the structural and functional characteristics of β-lactamases, their mode of action with drugs, and their alterations, in order to get a deeper understanding of the mechanistic basis for the development of antibiotic resistance. Methicillin-resistant Staphylococcus aureus (MRSA) has continued to evolve the *mecA* gene, encoding a novel penicillin-binding protein (PBP2a) with a low affinity for all β-lactams. This gene causes significant drug resistance, which has had major impacts on public health and safety (5).

**FIG 1:**
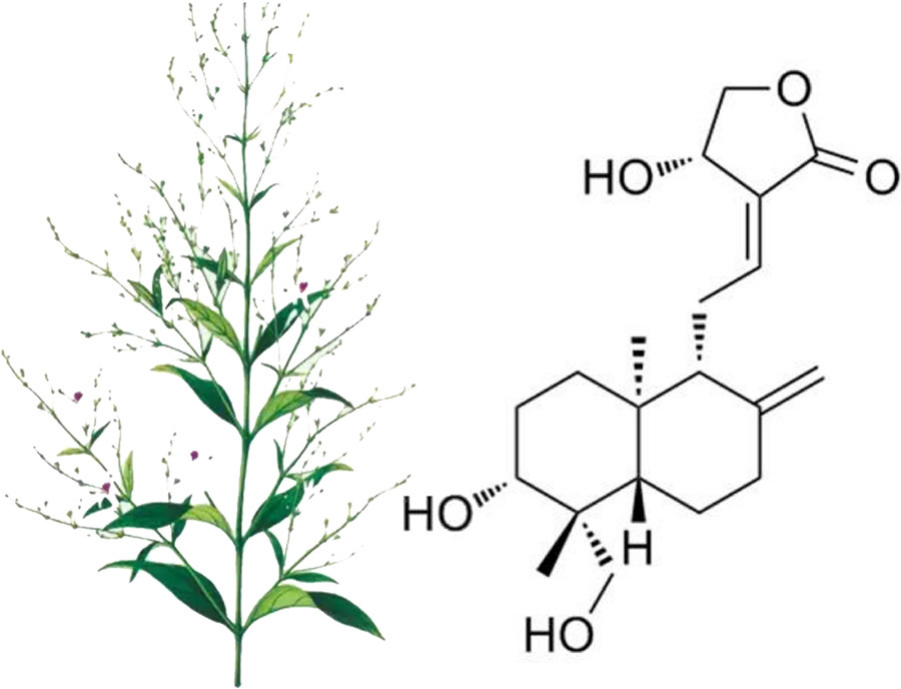
The medicinal herb Andrographis paniculata and Chemical structure of andrographolide (6).

Increasingly, alternatives to antibiotics are being used to mitigate the rapid onset and development of bacterial resistance, and the combination of natural bioactive compounds with traditional antibiotics has become an effective therapeutic strategy. As shown in Fig. 1, andrographolide (AP), derived from the medicinal herb Andrographis paniculata, acts as a natural diterpene lactone with a diverse pharmacological profile (6). In terms of its antibacterial activity, AP was reported to inhibit methicillin-sensitive S. aureus (MIC, 100 μg/mL) but had a particularly high MIC for MRSA (1,000 μg/mL), indicating that AP was not able to kill the MRSA strains with ease. Meanwhile, AP can effectively inhibit biofilm formation, and it increased the susceptibility of Pseudomonas aeruginosa to various antibiotics and suppressed the transcriptional abundance of the MexAB-OprM efflux pump gene (7, 8). However, it is unclear if AP could increase the susceptibility of resistant strains, such as the MRSA strain, to β-lactam antibiotics. Therefore, it is worth investigating the specific mechanisms linking AP and bacterial resistance.

Molecular dynamic simulation (MDS) at the atomic level provides high temporal and spatial resolution of molecular interactions, protein motions and conformational transitions. It exhaustively captures the subtle changes in the bonding process and also provides details regarding the additional energy involved in these interactions (9). However, these changes are difficult to observe with traditional biochemical experiments. It has been found in numerous drug resistance studies that even changes in residues that are far from the active site could still affect the hydrolytic activity of β-lactamases toward drugs through the interaction networks within the protein (10). Thus, MDS is able to capture the details of conformational changes and document the dynamic processes in a comprehensive manner. Meanwhile, the MDS approach also provides a new perspective on the molecular interactions and structure-function relationships (11). Furthermore, transcriptomic technologies that enable high-throughput analysis of differentially expressed genes (DEGs) provide systematic insights into the various aspects of intracellular changes and have been widely used for the discovery of new therapeutic targets and for uncovering the mechanism of action of various drugs. Combined with the vast amount of sequencing data publicly available today, it provides a novel avenue for deeper investigation of the molecular mechanisms of drugs (12).

In this study, we amplified the *blaZ* gene of S. aureus isolates from Ningxia Province by PCR and expressed and purified the β-lactamase BlaZ. We then analyzed the mode of binding of AP to BlaZ using fluorescence quenching, molecular docking, and MDS. The ability of AP to increase the susceptibility of MRSA strains to β-lactam antibiotics was confirmed by susceptibility tests. Furthermore, the mechanism of action of AP was analyzed by evaluating the gene regulation and morphological changes, and it was found that AP could increase antibiotics susceptibility by inhibiting the β-lactamase BlaZ, by downregulating the expression of critical resistance genes such as *mecA* and *blaZ*, and by disrupting the cell wall (CW) homeostasis. In addition, restoration of susceptibility to antibiotic could be achieved by inhibiting the global regulator SarA. The above results may provide a strong foundation for the discovery of novel inhibitors and more effective dosing strategies in the future.

## RESULTS

### Expression, purification, and structural analysis of the β-lactamase BlaZ.

**FIG 2:**
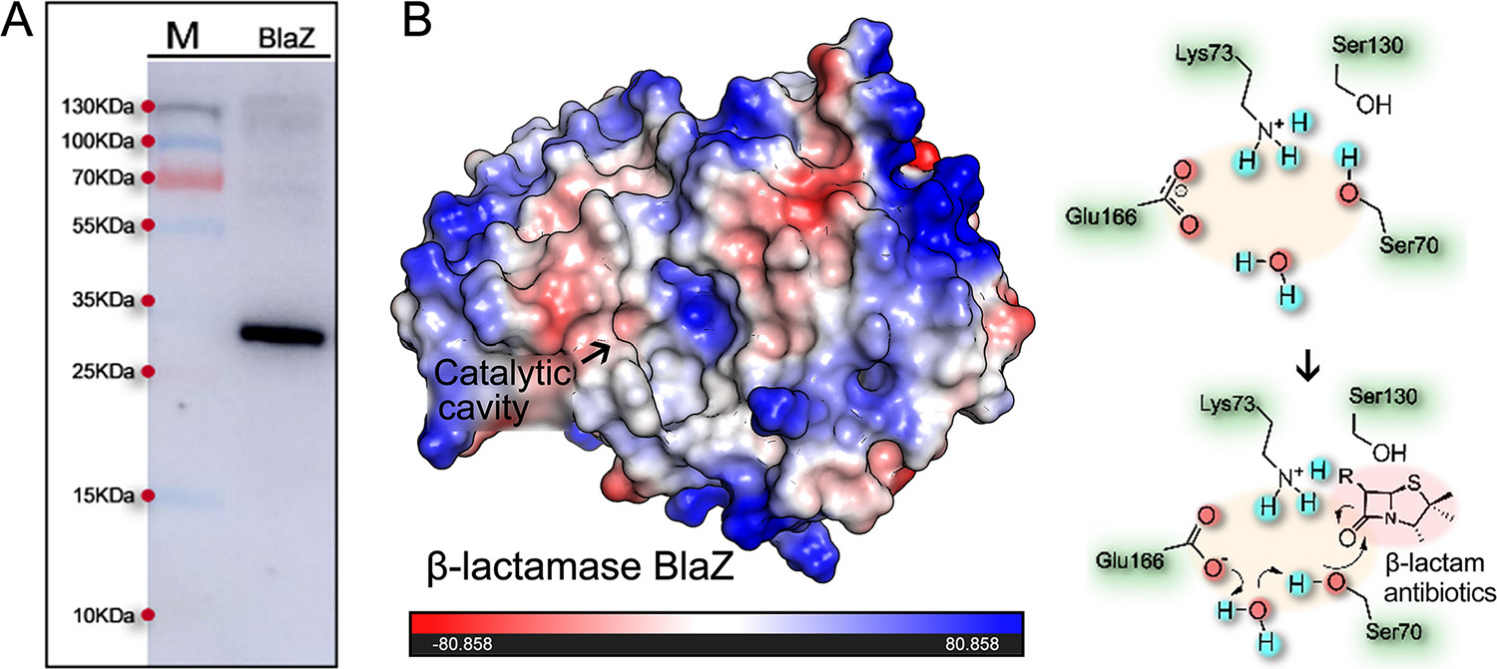
Expression purification and structural analysis of BlaZ. (A) Detection of BlaZ protein by Western blot assay. (B) Structural characterization of BlaZ. BlaZ has a catalytic cavity with SER70 as the active center and is involved in the hydrolysis process of β-lactam antibiotics. The surface electrostatic potential of the BlaZ was displayed. The red area represents the negative potential, the blue area represents the positive potential, and the catalytic cavity mainly presents the positive potential.

We amplified the *blaZ* gene with a fragment size of 861 bp and constructed a pET28a-*blaZ* recombinant plasmid. Successful expression of the BlaZ protein was detected by SDS-PAGE electrophoresis and Western blotting. Subsequently, the 6×His-BlaZ target protein was purified using nickel nitrilotriacetic acid (Ni-NTA) ion column chromatography. As shown in Fig. 2A, BlaZ protein with a molecular weight of 29.7 kDa was purified. Quantitative analysis of BlaZ showed that the concentration of the purified and concentrated protein was 4.04 × 10^−5 ^mol/L. Additionally, like most class A β-lactamases (serine β-lactamases), BlaZ is known to have a Ser70 at the center of the active site that plays a decisive role in the recognition and hydrolysis of the β-lactam antibiotics (13). There is a highly conserved sequence, Ser70-Thr71-Ser72-Lys73 (STSK), present at the bottom of the catalytic cavity (Fig. 2B). Furthermore, BlaZ has a conserved Glu residue at position 166. The OH and NH groups of Ser70 are known to participate in the formation of an oxygen anion hole and are involved in the acetylation and deacetylation processes of the hydrolysis reaction (14).

### Analysis of binding by fluorescence quenching.

**FIG 3:**
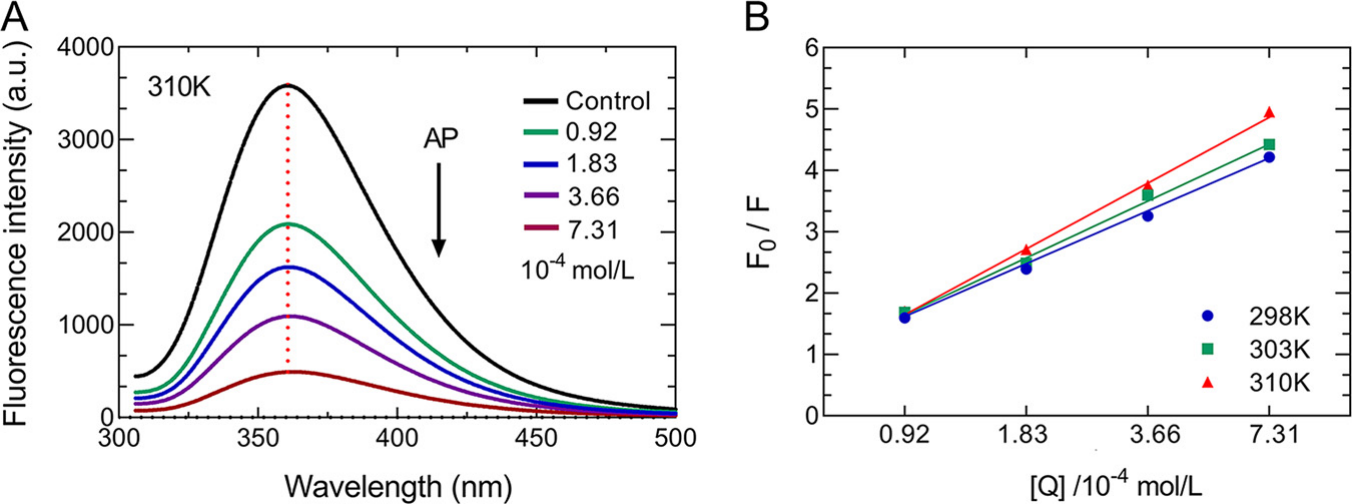
Effect of AP on BlaZ intrinsic fluorescence. (A) Fluorescence spectra of BlaZ in the presence of various concentrations of AP (310 K). (B) Stern-Volmer plot for BlaZ and AP.

The fluorescence quenching assay provides a unique perspective to study the possible binding interactions between AP and BlaZ protein. After incubation of purified BlaZ with 2.3 × 10^−5^ to 7.31 × 10^−4^ mol/L of AP, the change in the intrinsic fluorescence intensity of BlaZ was measured (298, 303, and 310 K). As shown in Fig. 3A, the excitation wavelength of BlaZ was 290 nm and its emission wavelength was 306.8 nm, whereby it could produce the strongest fluorescence at 354.4 nm (intensity: mean = 1,809 arbitrary units [AU], maximum = 2,004 AU), which gradually decreased after the peak, until the fluorescence disappeared after 500 nm. The intrinsic fluorescence intensity of BlaZ decreased continuously with the increase in AP concentration. In addition, BlaZ contains several Tyr residues and is predominantly characterized by these residues; the intrinsic fluorescence intensity of BlaZ decreased continuously with the increase of AP concentration, in the range of 9.2 × 10^−5^ to 7.32 × 10^−4^ mol/L, causing changes in the microenvironment of the Tyr due to BlaZ-AP interactions and ultimately causing a continuous decrease in intrinsic fluorescence. Furthermore, the values of *K*_sv_ (quenching constant) and *K*_q_ (rate constant in the quenching process) were calculated at three temperatures from the Stern-Volmer equation. When the values of *K*_q_ are larger than the maximum diffusive collisional quenching constant, 2 × 10^10^ L/mol/s (Table 1), it can be assumed that BlaZ forms a ground state complex with AP due to strong bonding (no excited-state formation) (15, 16). Therefore, this type of quenching can be considered static quenching (Fig. 3B).

**TABLE 1:** Stern-Volmer constants for the interaction of BlaZ with AP at three temperatures

Temp (K)	*K*_sv_ (10^3^ L/mol)	*K*_q_ (10^11^ L/mol/s)	*R* ^2^
298	6.82 ± 0.87	6.82	0.9953
303	7.09 ± 0.80	7.09	0.9842
310	7.21 ± 0.81	7.21	0.9917

### Analysis of the molecular binding mode.

**FIG 4:**
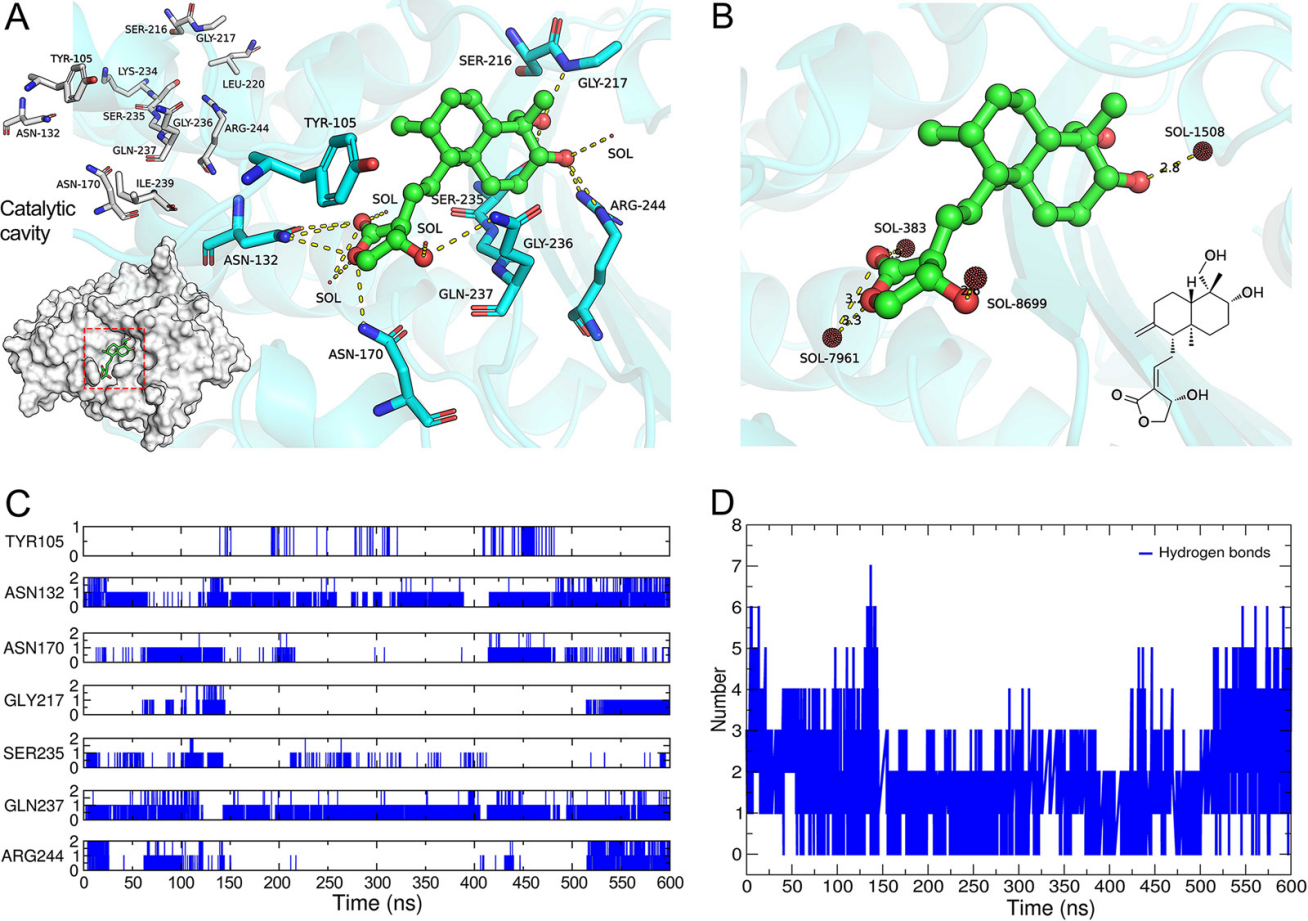
BlaZ and AP stable combination mode. (A) Conformation of binding of AP to residues inside the BlaZ catalytic cavity. (B) Discovery of solvent molecules involved in bond formation in molecular dynamics simulations. (C) Variation of hydrogen bonds formed by hot-spot residues and AP with simulation time. (D) Variation of overall hydrogen bonds during the simulation process.

Finally, the β-lactamase BlaZ was docked to AP and subsequently optimized by MDS at 600 ns to analyze the binding mode of the complex at steady state. It was worth noting that AP binds to the residues Asn132, Asn170, Gly217, Ser235, Gln237, and Arg244 in the catalytic cavity of BlaZ, forming an average of three hydrogen bonds (Fig. 4A). Meanwhile, a network of hydrogen bonds with the solvent molecules SOL383, -1508, -7961, and -8699, which entered the binding pocket, also participated in bonding and formed five hydrogen bonds (Fig. 4B). Analysis of the hydrogen bonds also revealed that three of these bonds were formed during the simulation. After 500 ns, Asn132, Gly217, Gln237, and Arg244 could form hydrogen bonds with AP consistently and stably, whereas Tyr105 did not stabilize the structure of AP through hydrogen bonds, but it did so through Van der Waals force (Fig. 4C and D). In short, hydrogen bonds were distributed around AP, making it stable in the catalytic cavity.

### Molecular dynamics simulation.

**FIG 5:**
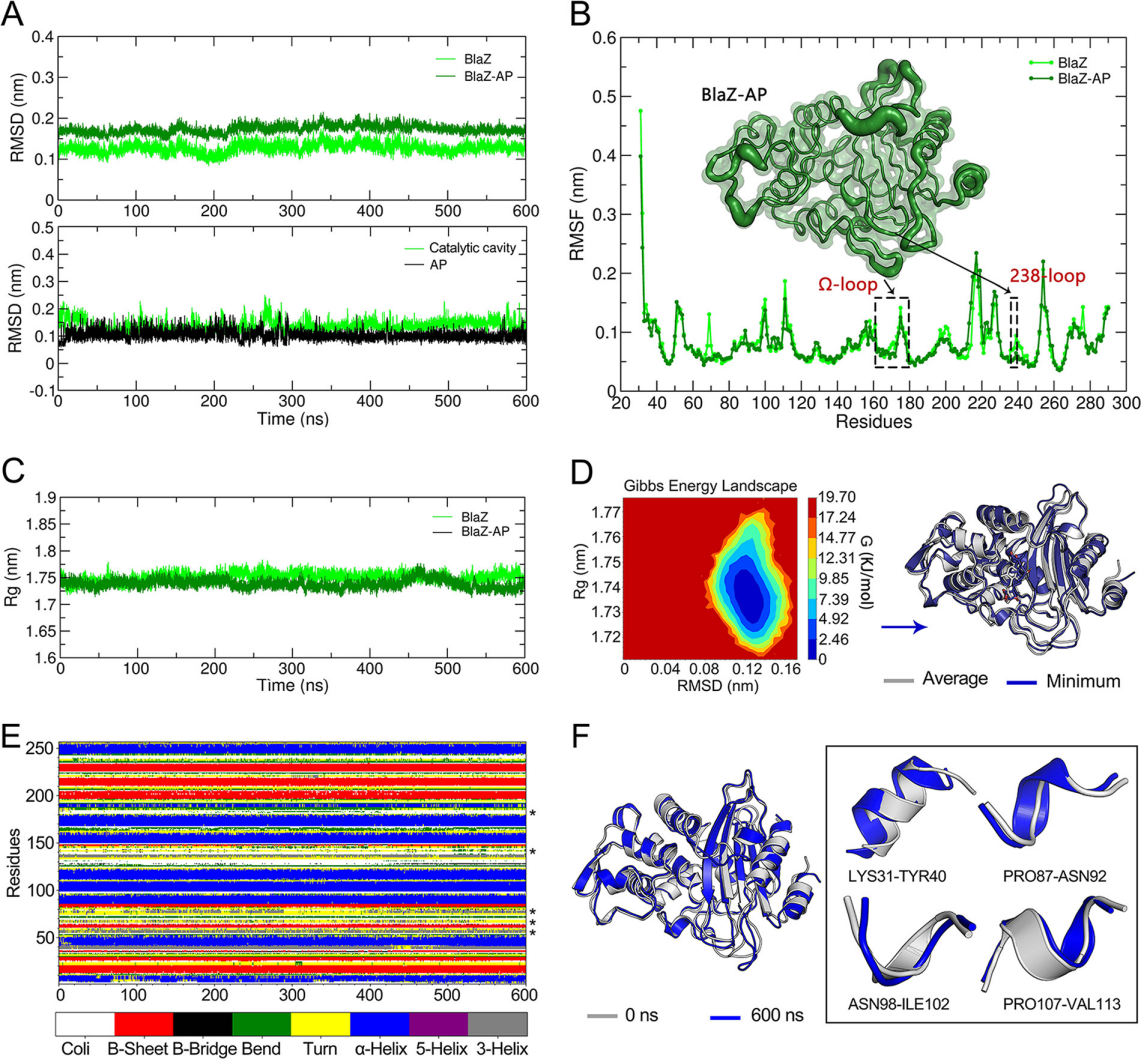
Molecular dynamics simulation of the BlaZ-AP. (A) RMSD plot of BlaZ-AP complex, BlaZ, catalytic cavity and AP. (B) RMSF plot of BlaZ-AP complex and BlaZ. The RMSF values are projected onto the tertiary structure of BlaZ and the fluctuations of Ω loop and 238 loop located at the inlet part of the catalytic chamber are demonstrated. (C) Rg plot of BlaZ-AP complex and BlaZ. (D) BlaZ-AP complex free energy landscape (FEL). The conformation of the minimum Gibbs free energy (dark blue) and the average conformation (gray) were superimposed to compare the conformational differences. (E) Variation of BlaZ secondary structure with simulation. (F) Conformational differences of BlaZ in BlaZ-AP complex at 0 ns and 600 ns.

To further characterize the changes and dynamics associated with the BlaZ-AP complex, MDS was performed over 600 ns. Subsequently, the root mean square deviation (RMSD) data were extracted sequentially for the BlaZ protein, AP, and the complex, in order to assess the stability of the conformation. As shown in Fig. 5A, the RMSD values of the complex ranged between 0.1142 and 0.2148 nm (mean = 0.1727 ± 0.0094 nm, maximum = 0.2148 nm). The values were higher than for apo-BlaZ (mean = 0.1233 ± 0.0112 nm, maximum = 0.1753 nm), but both showed higher stability. The RMSD values of AP (mean = 0.1233 ± 0.0367 nm, maximum = 0.2365 nm) and the hot-spot residues (mean = 0.1354 ± 0.0244 nm, maximum = 0.2599 nm) further reflected that the AP molecule could stably bind in the catalytic cavity of BlaZ. Meanwhile, the root mean square fluctuation (RMSF) of BlaZ before and after binding with AP was evaluated. It was found that the fluctuation of the whole protein decreased; that is, AP had a certain effect that led to conformational stabilization of BlaZ. The structural flexibility of the Ω loop (Asn161-Thr180) and the 238 loop (Als238-Arg44), which were at the entrance of the catalytic cavity, also appeared to be reduced (Fig. 5B). The radius of gyration (Rg) values for the BlaZ-AP complex were in the range of 1.7121 to 1.7767 nm, suggesting the lack of significant conformational changes in BlaZ (1.7173 to 1.7829 nm) after binding to AP (Fig. 5C). Additionally, the free energy landscape (FEL) reflected the relationship between the Gibbs free energy of the protein and RMSD as well as Rg when the energy-minimum conformation was superimposed on the average conformation. These structures were found to have a high degree of overlap, a finding that is consistent with the results from the RMSD and Rg assessments (Fig. 5D). As shown in Fig. 5E (asterisks), binding to AP caused changes in the secondary structure of BlaZ, with multiple transitions between β-turn, β-bend, and α-helix structures. The conformations of BlaZ in the BlaZ-AP complex at 0 ns and 600 ns were superimposed and compared, and four regions were found to show secondary structure transitions (Fig. 5F).

### Calculation of the free energy for binding.

**FIG 6:**
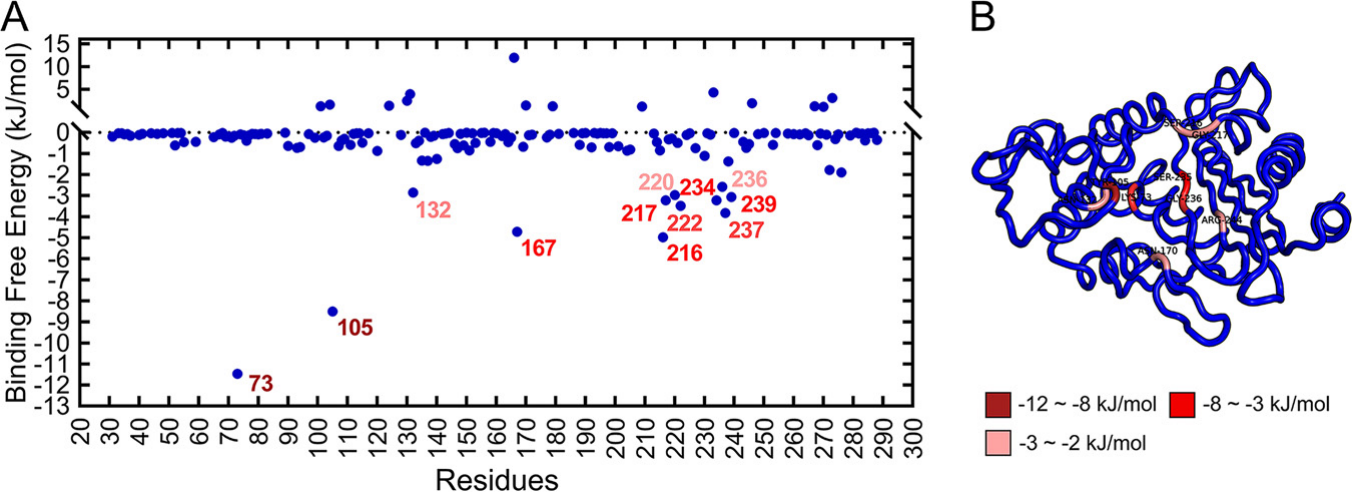
MM-PBSA binding free energy decomposition to each residue of BlaZ. (A) Contribution of hot-spot residues to binding free energy. (B) Residues with prominent binding free energy contributions are projected onto the protein structure. Dark red indicates energy contribution at −12 to −8 kJ/mol (Lys73 and Tyr105), red indicates energy at −8 to −3 kJ/mol (Lys216, Ile167, Gln237, Lys222, Gly217, Lys234, and Ile239), and light red indicates energy at −3 to −2 kJ/mol (Leu220, Asn132, and Gly236).

The free energy associated with the binding of AP to BlaZ was calculated using the Molecular mechanics Poisson–Boltzmann surface area (MM-PBSA) method, and the energy of each of the residues was decomposed to present the energy contribution of essential residues at the time of binding (17). The average free energy of binding, Δ*G*_bind_, was −111.317 ± 22.907 kJ/mol, and the Van der Waals energy (−148.545 ± 9.689 kJ/mol) occupied a larger proportion. In addition, the BlaZ-AP complex had an electrostatic energy of −74.67 ± 22.542 kJ/mol, a polar solvation energy of 128.357 ± 15.251 kJ/mol, and a the solvent accessible surface area (SASA) energy of −16.459 ± 0.743 kJ/mol. The Lys73 (−11.4576 ± 0.5676 kJ/mol) and Tyr105 (−8.4938 ± 0.3696 kJ/mol) residues made the most significant contributions to the free energy for binding. Moreover, the residues demonstrating free energies for binding below −2 kJ/mol were, in the following order, Lys216 (−4.9731 ± 0.4339 kJ/mol), Ile167 (−4.7158 ± 0.1525 kJ/mol), Gln237 (−3.8248 ± 0.8186 kJ/mol), Lys222 (−3.4845 ± 0.2507 kJ/mol), Gly217 (−3.215 ± 0.2553 kJ/mol), Lys234 (−3.211 ± 0.5994 kJ/mol), Ile239 (−3.0552 ± 0.1436 kJ/mol), Leu220 (−2.9663 ± 0.1093 kJ/mol), Asn132 (−2.8454 ± 0.5786 kJ/mol), and Gly236 (−2.5746 ± 0.2083 kJ/mol). The above hot-spot residues played an essential role in the binding process (Fig. 6).

### Combined disk test.

**FIG 7:**
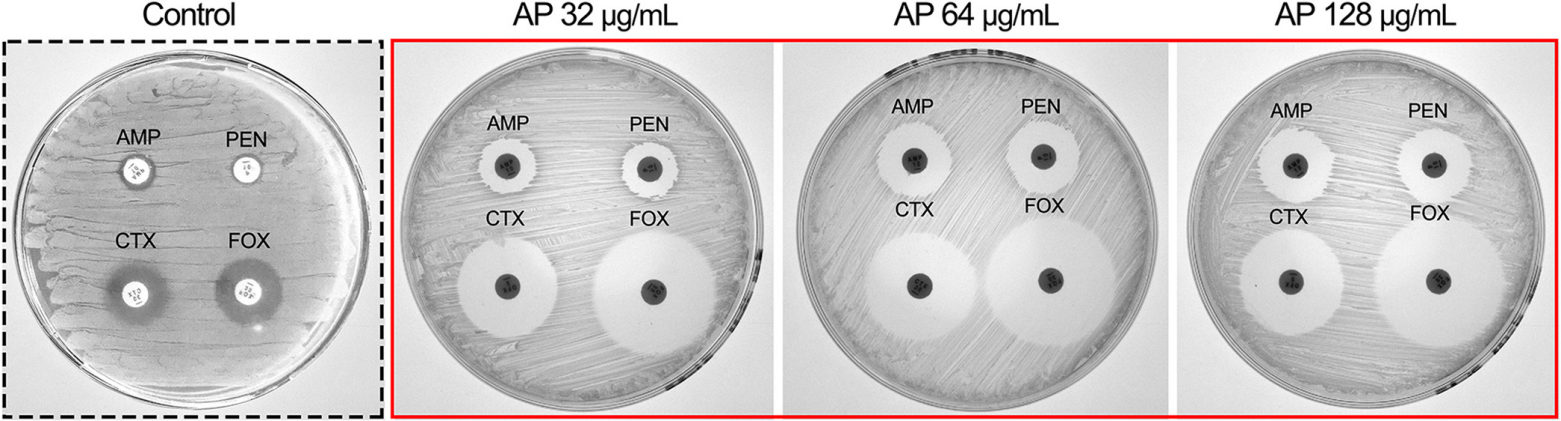
Effect of AP on the susceptibility of β-lactam antibiotics. AP at concentrations of 32, 64, and 128 μg/mL were used in combination with penicillin G (PEN), ampicillin (AMP), cefotaxime (CTX), and cefoxitin (FOX).

In the combined disk test, 32, 64, and 128 μg/mL AP was used in combination with β-lactam antibiotics (penicillin G, ampicillin, cefotaxime, and cefoxitin) to test the ability of AP to improve the susceptibility of the MRSA strain WLD10 to these antibiotics. Increasing concentrations of AP in the range of 0 to 64 μg/mL caused an increase in the inhibition zones of ampicillin (AMP) and penicillin G (PEN), and when AP concentration ranged between 64 μg/mL and 128 μg/mL, the inhibition zones no longer increased significantly, indicating a lack of dose-dependent inhibition. Meanwhile, there were more significant changes in the zone of inhibition with the cephalosporins cefotaxime (CTX) and cefoxitin (FOX) (Fig. 7). We speculated that this might be due to the presence of side chains with larger substituents in cephalosporins, which could have prevented their hydrolysis and entry into the catalytic cavity of BlaZ (18).

### Transmission electron microscopic observation of the cell wall.

**FIG 8:**
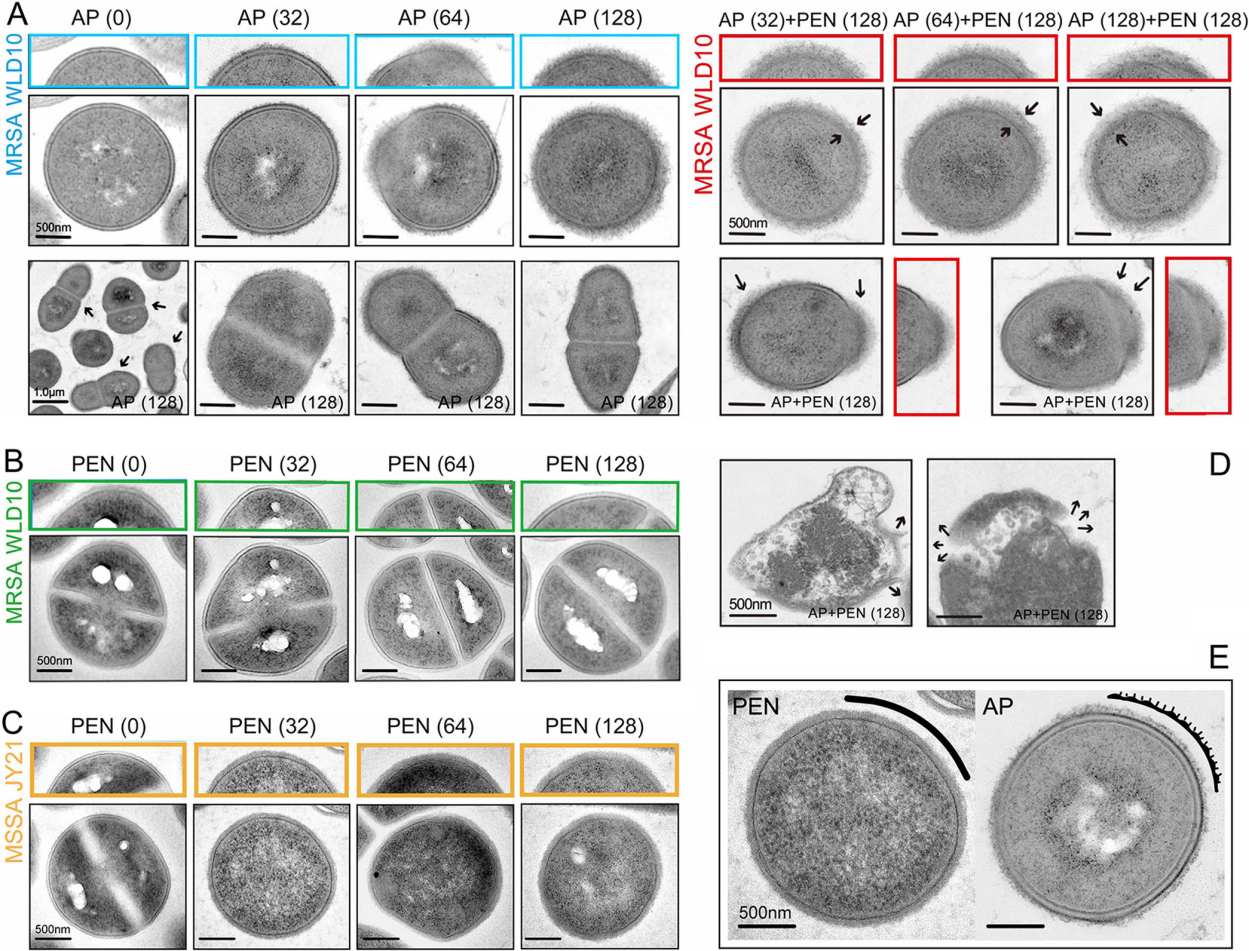
Effect of AP on cell wall of MRSA strain, determined by TEM. (A) Treatment of MRSA WLD10 with AP alone. (B) Treatment of WLD10 with PEN alone. (C) Treatment of MSSA JY21 with PEN alone. (D) Combination of different concentrations of AP and PEN for treatment of WLD10 strain. (E) Different effects of AP and PEN on the cell wall.

Observation under a transmission electron microscope (TEM) (Fig. 8A) showed that the control bacteria had a uniform round and oval morphology with clear CW borders and neat edges. The addition of AP at a concentration of 32 μg/mL caused the CW of MRSA WLD10 to turn rough at the edges but not thickened. However, when the concentration was further increased to 64 μg/mL, the CW turned further rough and fluffy, and the local outline became blurred, as some of the bacterial CW became depressed and mutilated. When the concentration of AP was further elevated to 128 μg/mL, the CW continued to thicken, the surface became obviously rough, and the outline could no longer be clearly distinguished. It should be emphasized that the CW was not uniformly thickened. It is worth noting that the diaphragm was normally formed, indicating that cell division was not inhibited despite the abnormal CW, which might also have reduced the stress to the MRSA. Moreover, PEN at concentrations of 32, 64, and 128 μg/mL did not cause visible changes in the morphology of the MRSA (Fig. 8B). Notably, it was confirmed that this intrinsically resistant bacterial strain was able to easily cope with the coercive pressure caused by the penicillins (19).

PEN, the traditional CW-active antibiotic, caused a significant thickening of the CW of MSSA JY21 at concentrations of 64 and 128 μg/mL. This adaptive resistance mechanism was achieved by the MSSA by increasing peptidoglycan (PG) synthesis and decreasing the cellular autolytic activity (Fig. 8C). However, the changes in the CW caused by AP and PEN were clearly different. As shown in Fig. 8E, instead of thickening, the surface of the CW became rough and fluffy after AP treatment, and when the AP concentration was increased to 128 μg/mL, the outline of the CW was no longer clear and had partially disappeared. This phenomenon reflected that the PG layer may have had errors in its cross-linking.

Furthermore, when 32 to 128 μg/mL of AP was added in combination with 128 μg/mL of PEN, the CW of the MRSA strain became fluffy, scattered, and rough in the field of view, except for a certain degree of thickening, and almost no bacteria were seen that had formed a diaphragm or were in the division stage and about to be constricted (Fig. 8D). This phenomenon reflected that when AP was used along with PEN, the original resistance mechanism of the MRSA could no longer cope well with the stress, resulting in the same concentration of PEN to cause CW abnormalities and structural damages in the MRSA strain. This caused a significant killing of the highly methicillin-resistant strain, consistent with the results from the antibiotic susceptibility tests (20).

### Transcriptomic sequencing analysis.

**FIG 9:**
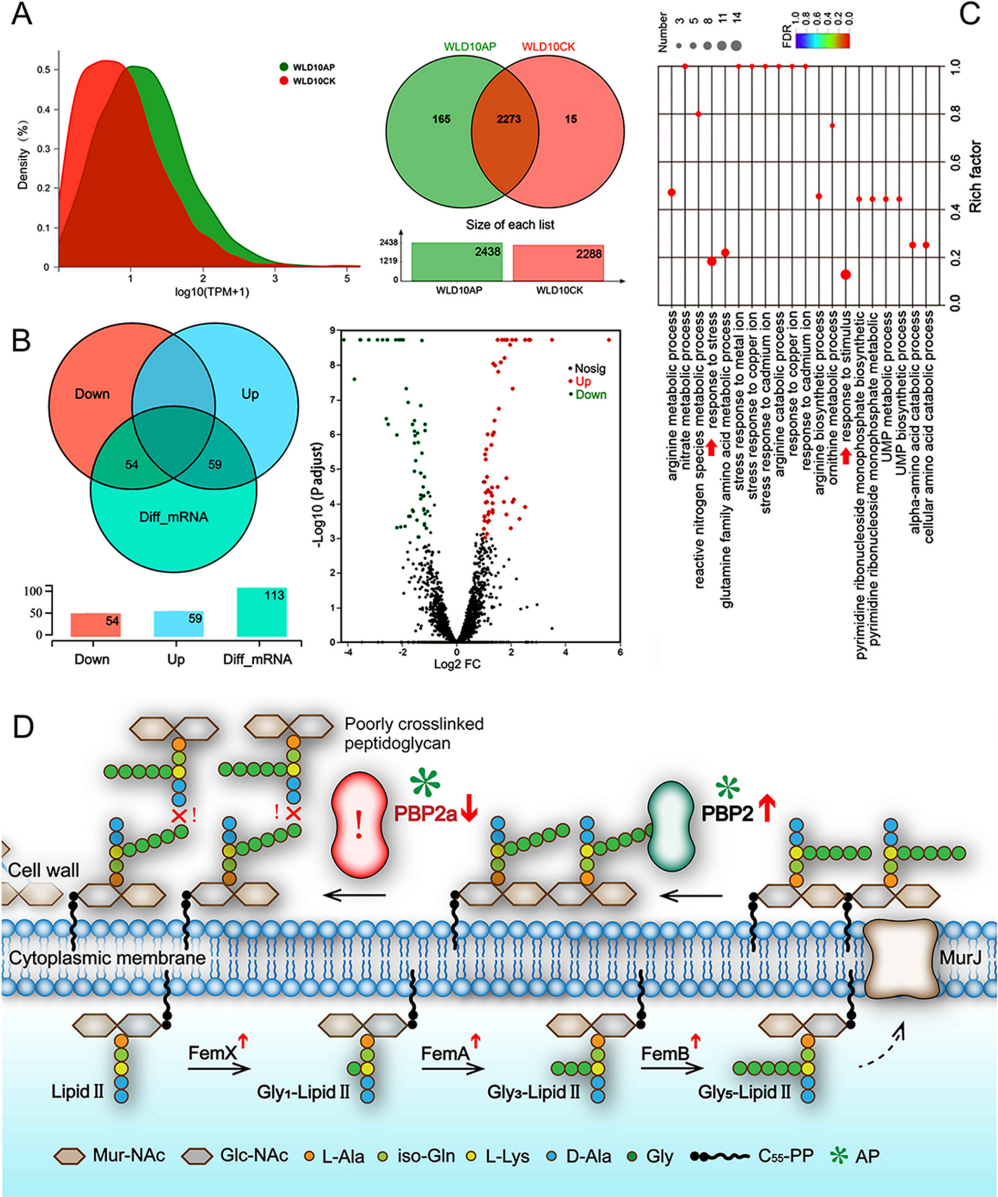
Transcriptomic sequencing results before and after AP treatment. (A) Analysis of gene expression in the control (WLD10CK) and experimental groups (WLD10AP). (B) Statistical analysis of DEGs. (C) GO enrichment analysis of DEGs. The vertical axis represents the description of the GO term, and the horizontal axis represents the Rich factor, which is the ratio of the number of genes annotated to the GO term in the gene set (sample number) to all genes (background number). The color of the dots corresponds to the different false discovery rate (FDR; *P* value corrected) ranges. The color of the dots corresponds to different FDR ranges, and the color indicates the significance of the enrichment. (D) Mechanistic diagram of the action of AP on the MRSA strain.

Statistically, a total of 2,288 genes were detected in the control group and 2,438 genes were detected in the AP-treated group, of which 2,273 genes were commonly expressed between the two groups, 165 were uniquely expressed in the treatment group, and 15 were uniquely expressed in the control group (Fig. 9A). This result indicated that AP increased the number of transcripts within the MRSA WLD10 strain by nearly 10-fold, suggesting that this set of uniquely expressed genes may most likely be involved in the stress response triggered by AP.

A total of 113 DEGs were detected in the experiment, of which 54 were downregulated and 59 were upregulated between the control and AP-treated group (Fig. 9B). Surprisingly, Gene Ontology (GO) enrichment analysis indicated that most of the DEGs were involved in the response to the stimulus and stress; multiple responses to ions also occurred. Meanwhile, the expression of the two-component regulatory system VraS/VraR showed differences wherein the genes mediated the response to CW damage and synthesize PG precursors and subsequently caused an increased expression of several genes such as *sgtB* and *murZ* (21, 22). Meanwhile, the helix-turn-helix (HTH)-type transcriptional regulator MgrA has been reported to block the activity of efflux pumps NorB and Tet38 and reduce cellular autolysis. Transcriptome analysis revealed that the upregulation of the *mgrA* gene may enhance the above processes and further improve the susceptibility of the MRSA strain to β-lactam antibiotics. The transcriptional repressor LexA is mainly involved in the response to DNA repair, through SOS response or recombination, and its coding gene, *lexA*, was found to be significantly upregulated in our study. Therefore, we hypothesized that AP may have caused damage to intracellular DNA (23, 24). Both *mecA* (down) and *mecI* (up) genes showed changes in their transcript levels, which consistently reduced the transcript levels of *mecA* in this study, thereby reducing the synthesis of PBP2a. The *blaI* gene is located upstream of the *blaZ* gene and is known to repress the transcription of *blaZ*. Increased transcription of the *blaI* gene has been reported to further weaken the resistance of the MRSA strains to β-lactam antibiotics (25). The sensor transducer genes *mecR1* and *blaR1* were upregulated, thereby reducing the resistance to penicillins (Fig. 9C).

The resistance factor FmtA, involved in mediating methicillin resistance, catalyzes the liberation of d-alanyl moieties present on wall teichoic acid (WTA) and lipoteichoic acid (LTA) (26). Methicillin resistance essential factors FemA and FemB catalyze the formation of the pentaglycine interpeptide bridge, which is characteristic of S. aureus (27). Generally, *fmtA*, *femA*, and *femB* are upregulated and enhance the physical barrier in response to AP-induced stimuli; however, the morphology of the CW had become mutilated and disorganized. The disordered and cross-linked CW was no longer stable enough to maintain its structure and resist antibiotic penetration. Meanwhile, stimulation with AP promoted an increase in PG synthesis, but observation under the TEM revealed a highly disturbed assembly and cross-linking of PG, preventing the normal localization of PBP2 and PBP2a (28). The abnormal assembly and cross-linking of PG might not allow β-lactam resistance to be expressed in the MRSA strain, despite normal PBP2a synthesis (Fig. 9D).

### Gene expression analysis.

**FIG 10:**
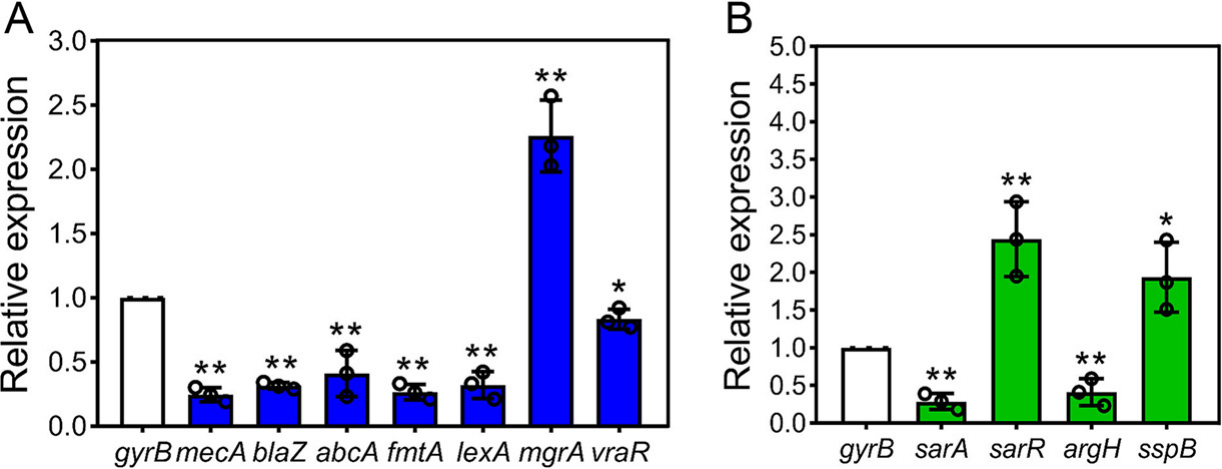
Results of qRT-PCR for critical resistance-associated genes. (A) Genes for β-lactam antibiotic resistance and stress response mechanisms. (B) Genes involved in the *sarA* repressor pathway. *, *P* < 0.05; **, *P* < 0.01.

As shown in Fig. 10A, there was a significant reduction in the transcript levels of both *mecA* (4.16-fold) and *blaZ* (3.22-fold), the core genes mediating antibiotic resistance in the MRSA strain. Meanwhile, the ABC transporter proteins are also known to be involved in the efflux of β-lactam antibiotics; the expression of *abcA* was found to be reduced by about 2.44-fold. A large number of genes related to ABC transporter permease and ATP-binding proteins were found to be altered in the transcriptome sequencing, with a general upregulation of metal-related ABC genes and a nonuniform trend in the remaining ABC-type genes. The two-component regulatory gene *vraR* was 1.20-fold downregulated. The *mgrA* gene was 2.26-fold upregulated, which might inhibit the autolysis activity, resulting in a failure to remove the old PG layer from the CW in a timely manner. There was a 3.13-fold downregulation in the expression of *lexA*, making it difficult to suppress the AP-induced DNA damage response. In addition, AP might also potentially suppress the transcription of *sarA* (Fig. 10B). The *sarA* gene was 3.45-fold downregulated, and another gene involved in the *sarA* regulatory system also showed changes in its transcript levels. SarR, a negative regulator of SarA, was 2.44-fold upregulated, reflecting the potential of AP in suppressing the global regulator SarA. Notably, the expression of *argH*, a gene upregulated by *sarA*, was 2.44-fold suppressed, and the expression of *sspB*, a gene downregulated by *sarA*, was 1.94-fold upregulated. The results from the quantitative reverse transcription-PCR (qRT-PCR) analysis were similar to the findings from the transcriptomic sequencing.

### GEO database mining and correlation analysis.

**FIG 11:**
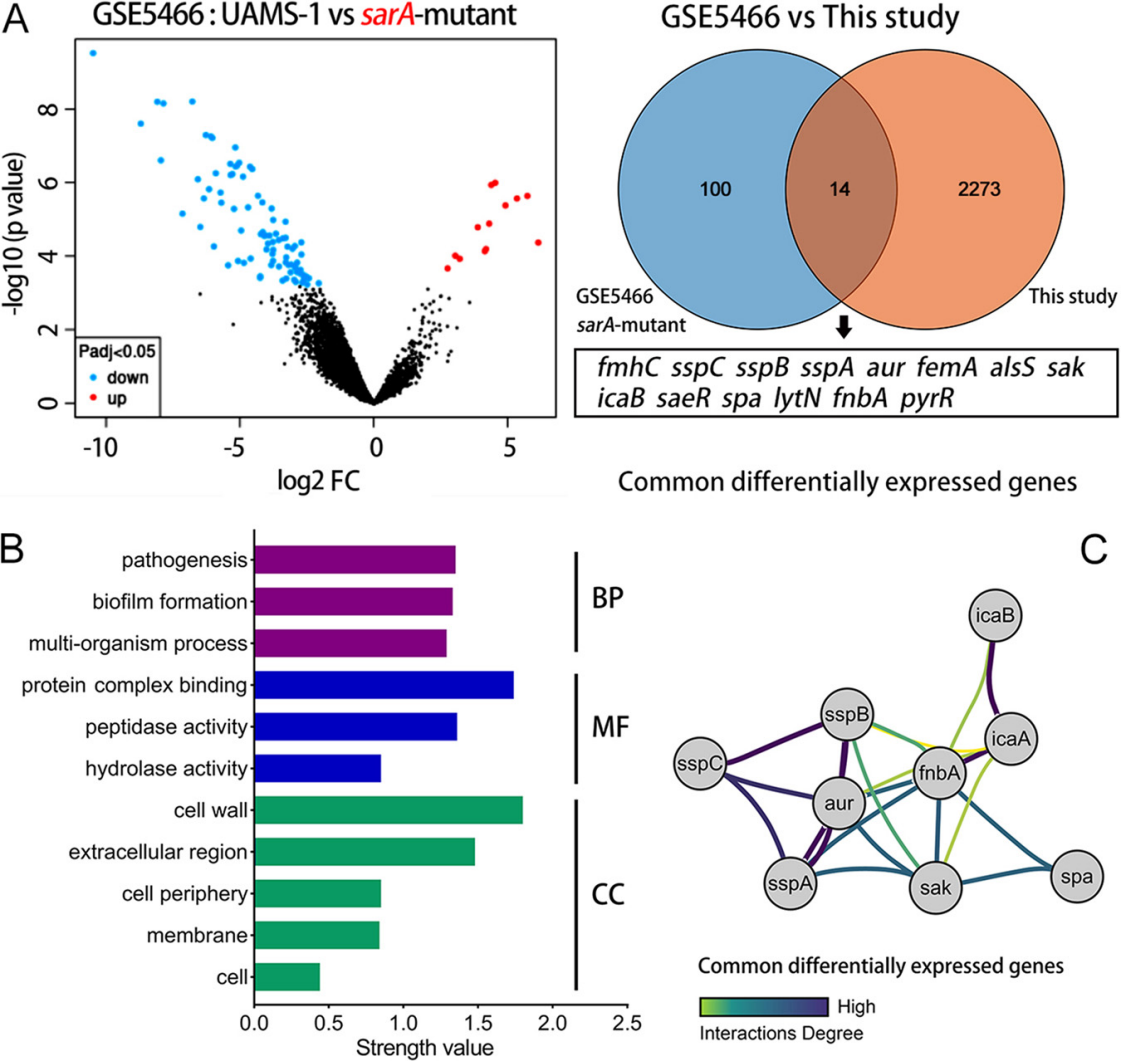
Association analysis of *sarA* gene mutation with this study. (A) Annotation and enrichment analysis of common differentially expressed genes. (B) GO enrichment analysis of common DEGs. BP, biological process; MF, molecular function; CC, cellular component. (C) Interaction network plot of common DEGs. The darker the color of the edges, the stronger their interaction.

Both transcriptomics and qRT-PCR analysis identified *mecA* transcript levels as being downregulated, possibly due to a direct effect of AP on the *mecI-mecR1* locus, suggesting a potential modality of regulation that is independent of *mecA*, which also enhances the sensitivity of the MRSA strain to β-lactam antibiotics. Recent research reported that mutations in *sarA* reduced the resistance of the MRSA strains to β-lactam antibiotics (29). It has also been shown that the regulatory factor SarA binds to and positively regulates the promoter region of the *mecA* gene (30). Based on these findings and the transcriptional changes identified by the transcriptomics analysis in this study, we hypothesized that there might also be a mechanism by which AP could downregulate *mecA* expression levels by repressing *sarA*. As shown in Fig. 11A, mining and analysis of the DEGs before and after the knockdown of the *sarA* gene in S. aureus UAMS-1, in the GSE5466 data set (31), resulted in a total of 100 DEGs, with 13 and 87 genes being up- and downregulated, respectively. There were significantly more downregulated genes than upregulated genes, which may be related to the fact that the SarA protein acts as a transcriptional regulator and acts primarily as a positive regulator of the downstream genes (32).

Meanwhile, the DEGs that emerged after mutation of the *sarA* gene were compared with those of this study, and 14 common DEGs were found, namely, *fmhC*, *sspC*, *sspB*, *sspA*, *aur*, *alsS*, *sak*, *femA*, *icaB*, *aseR*, *spa*, *lytN*, *fnbA*, and *pyrR*. Significantly, there was also consistency in the trends of gene variation with this study; all of the above findings pointed to the repression of *sarA* transcription, indicating some similarity between the effects mediated by AP treatment and mutations in the *sarA* gene. GO enrichment showed that the shared DEGs were mainly involved in pathogenesis, biofilm formation, and other processes. It was also noted that these genes were localized in the CW and membrane structures (Fig. 11B), which closely correlated with the changes in the CW as observed under the TEM. Furthermore, genes that were strongly associated with the biofilm showed a tendency to diminish its formation (Fig. 11C). The KEGG pathway was enriched in the *sams01503* and *sams05150* pathways and was mainly involved in the cationic antimicrobial peptide (CAMP) resistance and infections caused by S. aureus. This provided another confirmation that AP might also produce effects similar to that of *sarA* mutation, by suppressing the transcriptional levels of the *sarA* gene, ultimately restoring the susceptibility of MRSA strains to β-lactam antibiotics.

## DISCUSSION

The β-lactam antibiotics have long been considered effective in the treatment and prevention of infectious diseases caused by S. aureus and thus are widely used as first-line drugs in clinical practice. Nevertheless, the inappropriate use of these antibiotics has led to a rapid increase in drug-resistant strains and a rising rate of drug resistance, most typically represented by the emergence of MRSA strains, which seriously threatens the public health and safety of humans and animals (5). Meanwhile, several generations of β-lactam antibiotics have been updated, but they still cannot solve the prominent resistance problem of MRSA satisfactorily.

Although many researchers have achieved excellence in drug design and discovery, and artificial intelligence combined with computer-aided drug design has accelerated this process, discovery of novel backbones and critical targets is still a challenge (33, 34). Furthermore, the development of new drugs involves huge costs and years of continuous trials, and their subsequent inappropriate use may increase the onset and development of drug resistance and shorten the effective life of the drug (35). Therefore, using the available antibiotics properly is also a highly essential strategy. Natural active molecules such as AP, which have a multitarget effect and do not cause excessive stress on their own, can restore the susceptibility of intrinsically methicillin-resistant S. aureus strains to β-lactam antibiotics. Such compounds may reduce the amount of antibiotics used to some extent and offer an effective strategy to alleviate bacterial resistance—in short, making it possible to achieve the goal of “reducing dosage and increasing efficiency” of β-lactam antibiotics.

Our study also focuses on unraveling and elucidating the specific mechanism by which AP restores the susceptibility of MRSA strains to β-lactam antibiotics, as this is an issue that research has still not fully elucidated. Undeniably, production of β-lactamase is one of the primary mechanisms of resistance to β-lactam antibiotics. The ability of AP to bind stably within the catalytic cavity of β-lactamase BlaZ and to form an average of three hydrogen bonds over a time scale of 600 ns was shown by molecular docking and kinetic simulations. Liu et al. docked the natural compound magnolol with NDM-1 and formed two hydrogen bonds in a 100-ns simulation (36). An exhaustive understanding of the details of conformational changes in target molecular appears to be crucial in target-based drug discovery and design. Moreover, it is not hard to observe that both the type and distribution of functional groups of the inhibitor molecule itself affect the strength and stability of the binding. AP was able to form more hydrogen bonds and bind stably in the catalytic cavity due to the uniform distribution of OH in the end groups of the molecule, but the rigid planes formed by the intramolecular bicyclic parallelism also contributed to the instability of the binding. The simulations also revealed that the RMSD values for AP molecules in the 0- to 300-ns interval were slightly larger than after 300 ns, which may be related to the gradual formation of interactions such as π-π stacking in the plane after 300 ns.

Inhibition of the hydrolytic activity of β-lactamases alone does not fully restore the susceptibility to β-lactam antibiotics. With the help of the natural compound AP, which has multiple biological effects, the synthesis and cross-linking of the bacterial CW is altered, disrupting its homeostasis and allowing the CW-active antibiotics (β-lactams) to function better. As observed by transmission electron microscopy in this study, the morphological changes in the CW, which became rough, scattered, and fluffy after AP treatment, can be considered a bacterial response to the stimulus and activation of the CW stress stimulon (CWSS) mechanism to promote the synthesis of PG precursors (37). However, the transcription level and activity of PBP2a transpeptidase were inhibited, weakening the orderly cross-linking of peptidoglycan and significantly reducing the homeostasis of CW. It is interesting that the same concentration of PEN did not affect the CW morphology of the MRSA strains, and the changes in the CW of the MSSA strains induced by 64 and 128 μg/mL of PEN were different from those induced by AP treatment. In addition, the two-component regulatory system VraS/VraR may also be involved in CW metabolism and the regulation of CW-active antibiotic susceptibility, but not through the downregulation of *mecA* (29). Transcriptome sequencing also revealed that the upregulation of *vraR* gene attenuated the function of this system, which in turn increased the sensitivity of the MRSA strains to β-lactam antibiotics through a *mecA*-independent pathway.

Notably, AP also impaired the resistance of the MRSA strains to β-lactam antibiotics by repressing the Staphylococcus accessory regulator (SarA), which was also consistent with the findings from Wang et al. (30). Hypericin is known to enhance the activity of β-lactam antibiotics by reducing the transcriptional level of the *sarA* gene (30). SarA, a vital regulatory factor in S. aureus strains, regulates multiple virulence factors and multiple resistance mechanisms toward β-lactam antibiotics. Li et al. reported that the deletion of *sarA* gene in the MRSA strains reduced the MIC of *mecA* positive-MRSA strain to oxacillin (29). Those studies consistently revealed that SarA was involved in mediating resistance to β-lactam antibiotics and could broadly regulate virulence, resistance, and pathogenicity, and it was intensively studied as a key anti-S. aureus target. AP can cause a reduction in *sarA* and *mecA* transcript levels, raising the questions of whether SarA can bind to the promoter region of the *mecA* gene and activate its transcription and whether there is an atypical regulatory pathway. It is worthwhile to investigate these questions further. In addition, further studies exploring the interaction between AP and SarA are warranted to deepen our understanding of the antibiotic resistance mechanisms.

In summary, AP was able to stably bind to the β-lactamase BlaZ and impair its hydrolytic activity toward antibiotics. Simultaneously, it restored the susceptibility of the MRSA strains to the drug through mecA-dependent and independent pathways. Of note, the effect of AP on the CW of MRSA strains was different from that of PEN, and the changes in the CW phenotype observed in this study were consistent with a weakened function of PBP2a transpeptidase and stress response, disrupting the normal cross-linking and stability of the CW and also providing favorable conditions for the action of antibiotics. Interestingly, the DEGs that appeared after AP treatment had similarity to those after *sarA* deletion mutation, further supporting the notion that AP can affect drug sensitivity by regulating *sarA* transcription. The rational application of AP reduced the dosage of antibiotics and resensitized the intrinsically resistant strains to drugs with outstanding performance. Therefore, the current study demonstrates that AP effectively extended the therapeutic cycle of existing antibiotics and provides further theoretical references for the prevention and control of MRSA strains.

## MATERIALS AND METHODS

### β-Lactamase BlaZ purification and expression.

The genomic DNA of S. aureus isolated from Ningxia Province was used as a template to amplify *blaZ*, the gene encoding β-lactamase, by PCR, and the amplification products were sequenced for comparison. Subsequently, BamHI and Xhol I enzymatic (New England Biolabs [NEB]) cleavage sites and protective bases were introduced at both ends of the *blaZ* gene. The upstream primer was 5′-CGCGGATCCAAAGAGTTAAATGATTTA-3′, and the downstream primer was 5′-CCGCTCGAGTCAAAATTCCTTCTATACACT-3′ (restriction sites are underlined). The pET28a-*blaZ* recombinant plasmid was constructed and verified by double digestion; subsequently, gel recovery, ligation, and transformation were carried out. The positive clones were screened in LB agar (kanamycin resistant) and identified by sequencing, followed by the addition of isopropyl-β-d-thiogalactopyranoside (IPTG; Sigma-Aldrich) to a final concentration of 1 mM in the bacterial culture fluid. Furthermore, Escherichia coli BL21(DE3) organisms were collected at 12,000 rpm after 6 h of induction at 16°C, and the expression of the target protein was observed by SDS-PAGE. The BlaZ protein was purified by Ni ion affinity chromatography column. In addition, high concentrations of imidazole were removed by centrifugal filter devices (Millipore; 3 kDa), and the concentration was determined with a bicinchoninic acid (BCA) assay kit (Sigma-Aldrich).

### Western blotting.

After the transfer, the polyvinylidene difluoride (PVDF) film was removed and immersed in 5% (M/V) skim milk for 1 h, with slow shaking in a shaker for blocking. Then, the blocked PVDF membrane was submerged in the primary antibody (anti-His tag rabbit polyclonal antibody; Abbkine Scientific) overnight at 4°C and washed 3 times with 1× Tris-buffered saline–Tween (TBST) buffer for 10 min each time. Finally, the PVDF membrane was incubated with the secondary antibody (horseradish peroxidase [HRP]-conjugated goat anti-rabbit IgG; Abbkine Scientific) for 2 h at room temperature. The membrane was then developed by enhanced chemiluminescence (ECL) (Applygen Technologies).

### Fluorescence quenching.

Changes in the intensity of intrinsic fluorescence of proteins were measured by fluorescence spectrophotometry, enabling the analysis of the binding interaction between AP and BlaZ and the effect on protein conformation (38). The test conditions were as follows: excitation wavelength (λ_ex_) was 290 nm, emission wavelengths (λ_em_) were 306 nm (start) and 550 nm (end), and slit width was 5 nm (F-7000 FL; Hitachi). BlaZ (2 × 10^−5 ^mol/L) alone was used as a control group; the experimental groups were sequentially spiked with AP at concentrations ranging between 9.2 × 10^−5^ and 7.31 × 10^−4^ mol/L, and each sample was repeated three times to obtain the average value. The results were processed using FL solutions 2.1 for F-7000 and Origin v2019b software. The Stern-Volmer equation is as follows (39): *F*_0_/*F* = 1 + *K*_sv_[*Q*] = 1 + *K*_q_τ_0_[*Q*], where *F*_0_ is BlaZ fluorescence intensity, *F* is fluorescence intensity in the presence of quencher (AP), [*Q*] is the initial concentration of AP, *K*_sv_ is the quenching constant, *K*_q_ is the rate constant in the quenching process, and τ_0_ is the lifetime of elastase without AP (usually 10^−8^ s for macromolecules) (40).

### Molecular docking.

Molecular docking was performed to confirm that AP exhibited a sensitizing effect on MRSA toward β-lactam antibiotics, potentially by binding to β-lactamase and thus inhibiting its hydrolytic function. This experiment was carried out using the AutoDock Vina (41) and Smina (42) procedures to investigate the mode of binding of AP to BlaZ detected in this region (PDB ID 1BLC). Subsequently, the program AutoDock Tools 1.5.6 was used to hydrogenate the receptor protein BlaZ; the number of rotatable bonds of the ligand molecule and the grid box were set. The final docking boxes were determined to be as follows: center_x = 4.248, center_y = −5.035, and center_z = −13.347. After verification of the consistency of the above 2 docking procedures, the final docking results with both scoring and conformational fit were selected and visualized using PyMOL v2.4.0 (43).

### Molecular dynamics simulation and calculation of the free energy for binding.

All-atom molecular dynamics simulations were performed using the package GROMACS 2020.06 (44), using the BlaZ-AP complex after molecular docking as the initial conformation, in order to identify the mechanism of action and to verify the reliability of the binding model. The topology file of AP molecule was generated with the help of Antechamber and ACPYPE programs (45), the solventized box of the dodecahedron was chosen, and the nearest distance between the system boundary and the complex was set to 1.5 nm. The TIP3P water model was chosen, and 15 Cl^−^ ions were randomly added to the complex system according to the VERLET cutoff method to counterbalance the charge carried by the protein. Subsequently, the system was subjected to energy minimization, canonical ensemble (NVT) temperature control, and constant number of particles, pressure, and temperature (NPT) pressure control, so that the temperature of the system was 310 K and the pressure was constant at 101.325 kPa, and 600 ns free dynamic simulations were performed based on the above equilibrium system. The RMSD was used to measure the stability of the protein and the complex system by indicating the degree of molecular structure change. The RMSF and Rg reflected the effect of AP on BlaZ protein conformation; the number of hydrogen bonds formed between the receptor protein and the ligand molecule was analyzed as a function of the simulation time. In addition, the last 50-ns trajectories of the steady state were taken, and the free energy for binding was calculated by the MM-PBSA method (46). By definition in solution, we can write the binding free energy as follows: Δ*G*_bind_ = *G*_complex_ − (*G*_free-protein_ + *G*_free-ligand_). Then, the solvation free energy can be further decomposed into a polar and a nonpolar fraction: *G*_bind_ = *E*_gas_ − TS_gas_ + *G*_solvation_, where the solvation free energy can be further decomposed into a polar and a nonpolar fraction: *G*_solvation_ = *G*_polar_ + *G*_nonpolar_.

In the MM-PBSA method, the energy and entropy contributions of the gas phase were calculated according to the MM method, as follows: *E*_gas_ = *E*_MM_ = *E*_bond_ + *E*_angle_ + *E*_dihedral_ + *E*_vdw_ + *E*_coulomb_ and *S*_gas_ = *S*_MM_, where *E*_bond_, *E*_angle_, and *E*_dihedral_ correspond to bond, bond angle, and dihedral interactions, respectively, and *E*_vdw_ and *E*_coulomb_ represent van der Waals and Coulomb electrostatic interactions, respectively.

The solvation energy in the MM-PBSA method contains two components, the polar solvation energy and the nonpolar solvation energy. The polar solvation energy comes from the electrostatic interaction between the solute and solvent molecules and is calculated by using an implicit solvent model, wherein the solvent is considered a continuous medium, and the corresponding Poisson-Boltzmann equation is linearized and solved numerically: *G*_polar_ = *G*_PB_.

The nonpolar solvation free energy can be calculated based on the empirical surface area method and is therefore also known as the surface solvation energy. The calculation requires knowledge of the solvent-accessible surface area (*A*) of the molecule with two empirical parameters, γ and *b*: *G*_nonpolar_ = *G*_surface_ = γ*A* + *b*.

Combining the above terms yields the free energy equation for MM-PBSA: *G*_bind_ = *E*_MM_ − TS_MM_ + *G*_PB_ + *G*_surface_.

### Combined disk test.

WLD10 is a MRSA strain isolated from dairy cows with mastitis which exhibits more prominent resistance to penicillins than to cephalosporin antibiotics. The MICs were as follows: PEN MIC = 64 μg/mL, AMP MIC = 64 μg/mL, CTX MIC = 8 μg/mL, and FOX MIC = 4 μg/mL. Strain WLD10 was cultured to logarithmic growth phase and diluted to a concentration of 1 × 10^8^ CFU/mL (optical density at 600 nm [OD_600_] = 0.1). Simultaneously, the combined disk test (CDT) was performed according to Kali’s method (47), the bacterial suspension was evenly applied to Mueller-Hinton agar (MHA) with a sterile cotton swab, and the β-lactams disks (penicillin G, ampicillin, cefotaxime and cefoxitin; Oxoid, UK) were applied. The experimental groups were titrated with 10 μL of AP (32, 64, and 128 μg/mL) on disks. Finally, samples were subjected to constant incubation at 37°C for 24 h, and the diameters of inhibition zones were recorded.

### Observation by transmission electron microscopy.

The cultured bacteria were collected by centrifugation at 12,000 rpm and rinsed 3 times with phosphate-buffered saline (PBS) buffer (pH 7.0), and the supernatant was discarded. Four hundred microliters of glutaraldehyde was added at a concentration of 2.5%, and the samples were fixed at 4°C for 12 h. After staining with 1% osmium acid in a low-speed shaker for 1 h, the samples were treated with gradient dehydration using 50% to 90% ethanol, followed by sequential treatment with a mixture of acetone and epoxy resin (1:1) and (1:2). After the above steps were completed, 400 μL of 100% epoxy resin was added and polymerized in an oven at 60°C for 48 h. After embedding, frozen ultrathin sections of the samples were prepared. Finally, the prepared samples were observed and photographed under the TEM (120 kV; Hitachi HT 7800).

### Transcriptome sequencing analysis.

The MRSA WLD10 strain was cultured to logarithmic growth phase, and AP was added at a concentration of 128 μg/mL (1/8 MIC) to the treatment groups (3 biological replicates per group). The transcriptomic sequencing was performed by Shanghai Majorbio Bio-pharm Technology Co., Ltd. The high-quality sequences obtained after quality control were compared with the specified reference genome (Staphylococcus aureus strain MRSA252). After this, the Burrows-Wheeler method was used for mapping the sequencing results, and the index was generated by sorting and transforming the character matrix obtained after string conversion. The GO analysis was enriched and annotated mainly from the categories biological process (BP), molecular function (MF), and cell component (CC) to clarify the functions and biological significance of the DEGs that emerged after AP treatment. The regulatory and metabolic pathways involved in DEGs were analyzed by Kyoto Encyclopedia of Genes and Genomes (KEGG) enrichment. Genes with *P* values of <0.05 and log_2_ fold change (FC) of >1 were considered differentially expressed.

### qRT-PCR.

The total RNA extracted from S. aureus was used as a template for reverse transcription to generate a cDNA strand. We prepared the following reaction system: 2×qPCR mix enzyme, 7.5 μL; reverse transcription cDNA, 2.0 μL; and double-distilled water (ddH_2_O), 4.0 μL. qRT-PCR experiments were performed using the SYBR green master kit (Thermo Fisher Scientific). Using *gyrB* as a reference gene for standardized expression, the data were processed using the 2^−ΔΔ^*^CT^* method. The statistical differences between the samples were analyzed by *t* test, wherein a *P* value of <0.05 indicated statistical significance and a *P* value of <0.01 indicated a significant difference. The primers used in this study are shown in Table S3 in the supplemental material.

### GEO database mining and correlation analysis.

To further validate the above findings and identify the underlying mechanisms associated with AP-mediated susceptibility to β-lactam antibiotics, we retrieved the GSE5466 data set (31). This publicly available data set from NCBI Gene Expression Omnibus (GEO; https://www.ncbi.nlm.nih.gov/geo/) was highly relevant to our study. The downstream differential genes were extracted with the online tools GEO2R (https://www.ncbi.nlm.nih.gov/geo/info/geo2r.html) and R Studio v3.6.3. The DEGs shared between this study and the GSE5466 data set were analyzed and visualized using Cytoscape v3.82 (48) and TBtools software (49).
