# Supplementary material for: Insights into the Effects and Mechanism of Andrographolide-Mediated Recovery of Susceptibility of Methicillin-Resistant Staphylococcus aureus to β-Lactam Antibiotics
Source: Microbiol Spectr. 2023 Jan 5;11(1):e02978-22. doi: 10.1128/spectrum.02978-22 (PMC9927479; doi:10.1128/spectrum.02978-22)
Supplement: Supplemental file 1 — Supplemental material. Download spectrum.02978-22-s0001.pdf, PDF file, 0.2 MB [file spectrum.02978-22-s0001.pdf]

Manuscript title: Insights into the effects and mechanism of andrographolide mediated recovery of susceptibility of methicillin-resistant *Staphylococcus aureus* to β-lactam antibiotics

Supplementary Table. 3

Primers used in the qRT-PCR.

Genes	Primer sequences（5'→3'）	Fragment size / bp	Annealing temperature / ℃
gyrB	F: TCCTGCGACAAGTGAATTGAA<br>R: AAAGCTGGACGCACAGTGTCT	198	60
mecA	F: GTTAGATTGGGATCATAGCGTCATT<br>R:TGCCTAATCTCATATGTGTTCCTGTAT	210	59
blaZ	F: AAATATGTAGGAAAAGATATC<br>R: TATCTTTTTTGCTCTTTGGTG	208	57
abcA	F: TCGTCCAACAGCAACAGATGA<br>R: TCATATCCCTGTGGCAAGTTCA	205	60
mgrA	F: TAATTTAAAAGAACAGCTATGC<br>R: CAGTATCGAGTGCTAATTCAGT	185	57
lexA	F: CACCAACACGGGCTGAAAT<br>R: TGGCAATCCTTCTGGCTCT	161	59
sarA	F: CAATTAGCTTTGAAGAATTCGCT<br>R: CGAAGTAATCTTCTTGAGATAAAAT	190	60
fmtA	F: AGCACACAACAAGCGAGATAAC<br>R: GCATTTGATGTACCACCAGCAT	171	57
vraR	F: TGAGTCGTCGCTTCTACACCAT<br>R: ATTGCCAAAGCCCATGAGTT	195	57
pbpB	F: AGCGATTGATGGTGATACGG<br>R: TTGCTTCAGGGCCATATTTC	217	60
sarR	F: ATTTAACTAAAGCTTTACAAAA<br>R: GTTCAATGTTATTAAACGCGGT	190	59
argH	F: ATGAATTTAGCTTCGTCTGTGG<br>R: CTGCAGCCTTAAGTGGTACC	211	60
sspB	F: TACTGAATCCTGCACACCATGAG<br>R: CCACTACCTGGTAATGTAAAAGA	205	60
